# Solubility Data of the Bioactive Compound Piperine in (Transcutol + Water) Mixtures: Computational Modeling, Hansen Solubility Parameters and Mixing Thermodynamic Parameters

**DOI:** 10.3390/molecules25122743

**Published:** 2020-06-13

**Authors:** Faiyaz Shakeel, Nazrul Haq, Sultan Alshehri

**Affiliations:** Department of Pharmaceutics, College of Pharmacy, King Saud University, P.O. Box 2457, Riyadh 11451, Saudi Arabia; faiyazs@fastmail.fm (F.S.); nazrulhaq59@gmail.com (N.H.)

**Keywords:** activity coefficient model, bioactive compound, piperine, solubility, solution thermodynamics, Transcutol

## Abstract

The solubility values and thermodynamic parameters of a natural phytomedicine/nutrient piperine (PPN) in Transcutol-HP (THP) + water combinations were determined. The mole fraction solubilities (*x*_e_) of PPN in THP + water combinations were recorded at *T* = 298.2–318.2 K and *p* = 0.1 MPa by the shake flask method. Hansen solubility parameters (HSPs) of PPN, pure THP, pure water and THP + water mixtures free of PPN were also computed. The *x*_e_ values of PPN were correlated well with “Apelblat, Van’t Hoff, Yalkowsky–Roseman, Jouyban–Acree and Jouyban–Acree–Van’t Hoff” models with root mean square deviations of < 2.0%. The maximum and minimum *x*_e_ value of PPN was found in pure THP (9.10 × 10^−2^ at *T* = 318.2 K) and pure water (1.03 × 10^−5^ at *T* = 298.2 K), respectively. In addition, HSP of PPN was observed more closed with that of pure THP. The thermodynamic parameters of PPN were obtained using the activity coefficient model. The results showed an endothermic dissolution of PPN at *m* = 0.6–1.0 in comparison to other THP + water combinations studied. In addition, PPN dissolution was recorded as entropy-driven at *m* = 0.8–1.0 compared with other THP + water mixtures evaluated.

## 1. Introduction

**Figure 1:**
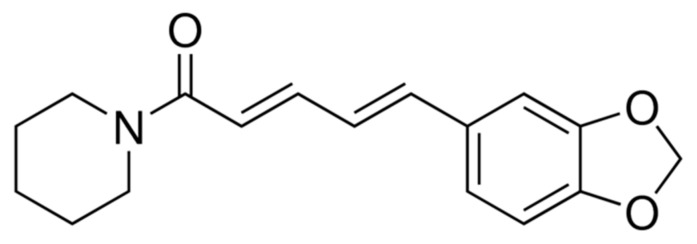
Chemical structure of piperine (PPN).

Piperine (PPN; Figure 1) is a bioactive alkaloidal phytomedicine/nutrient that is present in the fruits and roots of *Piper nigrum* and *Piper longum* [1,2]. The pungency and bitter taste of pepper are due to the presence of PPN [2]. PPN is a potent bioactive compound, which has been reported to have several therapeutic activities including “anti-metastatic [3], enzyme activity stimulator [4], antimicrobial [5], antifertility [6], hepatoprotective [7], antiulcer [8], antiamoebic [9], antidiarrheal [10], anti-fibrotic [11], antifungal [12], acaricidal [13], anti-inflammatory [14,15], antioxidant [2,16,17] and anticancer activity [2,18]”. In addition, PPN has also been reported as a permeation and bioavailability enhancer for several weakly soluble drugs as well as nutrients [1,2,19,20,21]. The solubilization of phytomedicines/nutrients in co-solvent–water mixtures had significant role in their “isolation, extraction, purification, recrystallization, drug discovery and dosage form design” [22,23,24]. Therefore, the solubilization of PPN in co-solvent–water mixtures should be studied in order to obtain its application in pharmaceutical and food industries. Transcutol-HP (THP) is a potential co-solvent that is miscible with all proportions of water [24,25]. Its potential in increasing the solubilization of several poorly soluble bioactive compounds including vanillin, reserpine, sinapic acid and apigenin has been very well reported in the literature [24,25,26,27]. Some formulation technologies including solid dosage forms [28,29], emulsion/self-emulsifying formulations [30,31,32], nanomedicine-based drug delivery systems [33,34,35,36] and solid dispersion technology [37] have been studied in order to enhance solubility and bioactivity/bioavailability of PPN. The solubility of PPN in pure solvents including pure water, pure propylene glycol (PG), pure polyethylene glycol-400 (PEG-400) and pure THP at ambient temperature was reported elsewhere [1,30,31]. The solubility and mixing thermodynamic parameters of PPN in twelve different pure solvents including “water, methanol, ethanol, isopropanol, 1-butanol, 2-butanol, ethylene glycol, PG, PEG-400, ethyl acetate, dimethyl sulfoxide and THP” at “*T* = 298.2–318.2 K” and “*p* = 0.1 MPa” have also been reported [38]. The solubility data of PPN in water–ethanol and water–surfactant mixtures was also found elsewhere [39,40,41]. The solubility values of PPN in super critical carbon dioxide (CO_2_) and near critical CO_2_ at different temperatures has also been reported elsewhere [42]. So far, there is no report on PPN solubilization in “THP + water” mixtures at “*T* = 298.2–318.2 K” and “*p* = 0.1 MPa”. Therefore, in this research, the solubility profile of PPN in various “THP + water” mixtures, including pure water and pure THP at “*T* = 298.2–318.2 K” and “*p* = 0.1 MPa” is studied and reported. Mixing thermodynamic parameters of PPN are also computed and reported using an activity coefficient model. The solubility values of PPN reported in this research could be beneficial in “isolation, extraction, purification, recrystallization, drug discovery, pre-formulation studies and dosage form design” of PPN. 

## 2. Results and Discussion

### 2.1. Experimental Solubility Values of PPN and Literature Comparison

The “mole fraction solubility (*x*_e_)” values of PPN in various “THP + water” combinations including pure water and pure THP at “*T* = 298.2–318.2 K’ and “*p* = 0.1 MPa” are summarized in Table 1. The solubility values of PPN in pure water and pure THP have been reported [38]. However, its solubility values in “THP + water” mixtures have not been reported elsewhere so far. 

**Figure 2:**
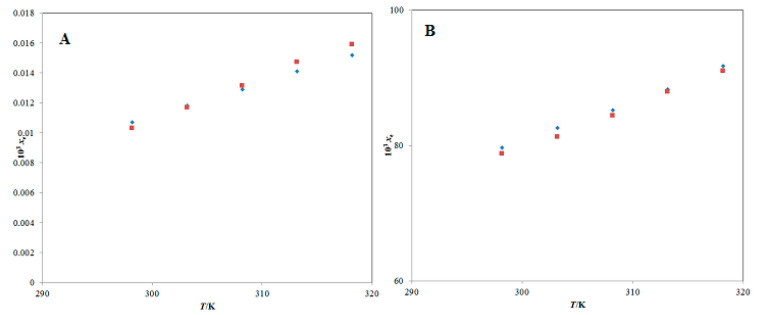
Comparison of mole fraction solubility of PPN in (**A**) pure water and (**B**) pure Transcutol-HP (THP) with reported solubilities at “*T* = 298.2 K to 318.2 K”; the symbol 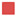 shows the experimental mole fraction solubility of PPN in (**A**) pure water and (**B**) pure THP, and the symbol 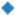 shows the reported solubilities of PPN in (**A**) pure water and (**B**) pure THP taken from reference [38].

The solubility of PPN in pure water at “*T* = 298.2 K” was recorded as 0.164 mg g^−1^ (equivalent to *x*_e_ = 1.04 × 10^−5^) and 10 µg g^−1^ (equivalent to *x*_e_ = 6.31 × 10^−7^) by Shao et al. and Veerareddy et al., respectively [30,31]. In addition, the solubility of PPN in water at “*T* = 291.2 K” was obtained as 40 µg g^−1^ (equivalent to *x*_e_ = 2.53 × 10^−6^) by another report [1]. The *x*_e_ value of PPN in pure water at “*T* = 298.2 K” was calculated as 1.03 × 10^−5^ in the current research (Table 1). The solubility of PPN in pure THP at “*T* = 298.2 K” was obtained as 185.29 mg g^−1^ (equivalent to *x*_e_ = 8.01 × 10^−2^) [31]. The *x*_e_ value of PPN in pure THP at “*T* = 298.2 K” was calculated as 7.88 × 10^−2^ in the current research (Table 1). The *x*_e_ values of PPN in pure water and pure THP obtained in the current research were found to be very close to those reported by Shao et al. [31]. However, the *x*_e_ value of PPN in pure water obtained in the current research was found to have deviated much from those reported by Veerareddy et al. and Vasavirama and Upender [1,30]. This deviation could be due to the variation in shaking speed, equilibrium time and analysis method of PPN [1,30,38]. The solubility values of PPN in pure water and pure THP at five different temperatures, i.e., “*T* = 298.2–318.2 K” have also been reported [38]. The graphical comparison between *x*_e_ and literature solubility values of PPN in pure water and pure THP at “*T* = 298.2–318.2 K” are summarized in Figure 2A,B, respectively. The data summarized in Figure 2A,B suggested an excellent correlation of *x*_e_ values of PPN with the literature solubility data of PPN in pure water and pure THP at “*T* = 298.2–318.2 K”. Overall, the experimental solubility values of PPN obtained in the current research were found in good agreement with those reported in the literature. The reliability of the proposed method of solubility measurement was verified by obtaining the *x*_e_ values of another phytomedicine/nutraceutical apigenin in pure THP at *T* = 298.2 K and *T* = 318.2 K. The *x*_e_ value of apigenin in pure THP at *T* = 298.2 K and *T* = 318.2 K was found as 3.36 × 10^−1^ and 3.82 × 10^−1^, respectively, in the literature [27]. The *x*_e_ value of apigenin in pure THP at *T* = 298.2 K and *T* = 318.2 K was determined as 3.33 × 10^−1^ and 3.84 × 10^−1^, respectively, in the current research. These results suggested that the *x*_e_ value of apigenin in pure THP obtained using the current technique was very close to those reported in the literature [27]. Therefore, the present technique of solubility measurement was reliable for the solubility determination of PPN.

**Figure 3:**
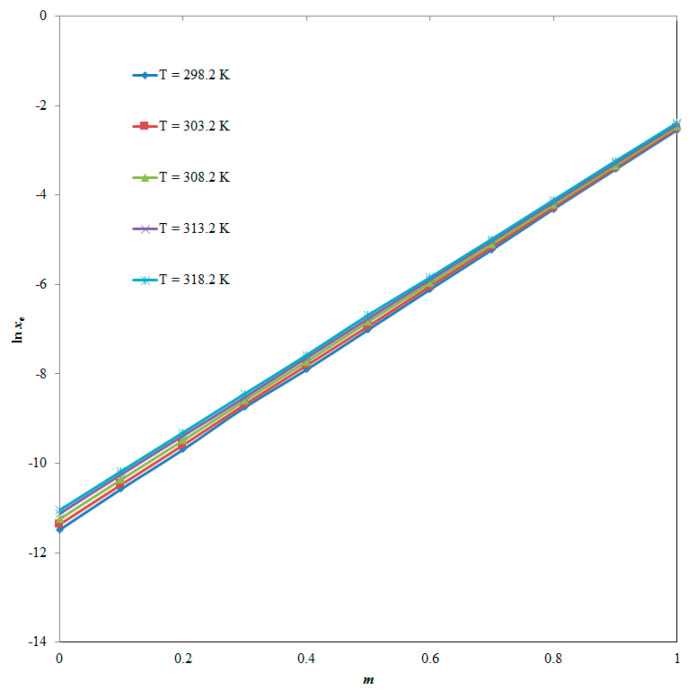
Effect of mass fraction of THP (*m*) on solubility of PPN at “*T* = 298.2–318.2 K”.

As per the results summarized in Table 1, the *x*_e_ values of PPN were found to increase with increases in both THP mass fraction (*m*) in various “THP + water” combinations and temperature, and therefore the minimum *x*_e_ value of PPN was obtained in pure water (*x*_e_ = 1.03 × 10^−5^) at “*T* = 298.2 K”, and the maximum *x*_e_ value of PPN was observed in pure THP (*x*_e_ = 9.10 × 10^−2^) at “*T* = 318.2 K”. The maximum *x*_e_ value of PPN in pure THP could be due to the lower polarity and low Hansen solubility parameter (HSP) of THP in comparison to high polarity and higher HSP of water [25,26]. The impact of *m* value of THP on PPN solubility at “*T* = 298.15–318.15 K” is summarized in Figure 3. 

As per these results, the PPN solubility was found to increase linearly with increases in *m* values of THP at all five temperatures studied. It was also observed that the *x*_e_ values of PPN were significantly enhanced from pure water to pure THP, and therefore THP could be utilized as an excellent co-solvent in PPN solubility enhancement.

### 2.2. Hansen Solubility Parameters (HSPs)

The results of HSPs of different “THP + water” systems free of PPN are tabulated in Supplementary Materials Table S1. The HSP (*δ*) value of PPN was computed as 22.30 MPa^1/2^. The HSP value for pure THP (*δ*_1_) and pure water (*δ*_2_) were computed as 21.40 and 47.80 MPa^1/2^, respectively. The HSP values for various “THP + water” mixtures free of PPN (*δ*_mix_) were computed as 24.04–45.16 MPa^1/2^. As per the data recorded, the HSP value of pure THP (*δ*_2_ = 21.40 MPa^1/2^) and “THP + water” mixtures (at *m* = 0.9; *δ*_mix_ = 24.04 MPa^1/2^) were found to close to that of PPN (*δ* = 22.30 MPa^1/2^). The *x*_e_ values of PPN were also obtained at the maximum in pure THP and at *m* = 0.9 of THP in “THP + water” mixtures. Hence, the obtained solubility data of PPN in various “THP + water” mixtures was in good agreement with their HSPs

### 2.3. Mixing Thermodynamic Parameters of PPN Solution

The computed values of various mixing thermodynamic parameters such as “mixing Gibbs energy (Δ_mix_*G*), mixing enthalpy (Δ_mix_*H*) and mixing entropy (Δ_mix_*S*)” along with activity coefficients (*γ*_i_) for PPN in different “THP + water” combinations including pure water and pure THP are given in Supplementary Materials Table S2. The Δ_mix_*G* values for PPN at *m* = 0.6–1.0 were found as negative values, which decreased with the increase in temperature. Hence, PPN dissolution was proposed as endothermic at *m* = 0.6–1.0. The Δ_mix_*S* values for PPN at *m* = 0.8–1.0 were found as positive values, which also decreased with increases in temperature. Therefore, PPN dissolution was proposed as entropy-driven at *m* = 0.8–1.0. The Δ_mix_*H* values for PPN were found as negative values in most of the “THP + water” combinations studied. 

### 2.4. Solute–Solvent Molecular Interactions

The data of *γ*_i_ for PPN in different “THP + water” combinations including pure water and pure THP at “*T* = 298.2–318.2 K” is summarized in Table 2. The *γ*_i_ value obtained for PPN was highest in pure water at all five temperatures studied. However, the *γ*_i_ value obtained for PPN was lowest in pure THP at all five temperatures. The highest *γ*_i_ value for PPN in pure water could be possible due to the lowest *x*_e_ value of PPN in pure water. As per these results, the *γ*_i_ value for PPN was found to increase with increases in temperature in all “THP + water” mixtures studied. Based on these results, the maximum solute–solvent interactions were considered in PPN–THP compared with PPN–water. 

### 2.5. Modeling of PPN Solubility

The solubility data obtained for PPN was correlated using “Van’t Hoff, Apelblat, Yalkowsky–Roseman, Jouyban–Acree and Jouyban–Acree–Van’t Hoff” models [26,43,44,45,46,47,48]. Results of the “Van’t Hoff model” for PPN in different “THP + water” mixtures including pure water and pure THP are summarized in Table 3. 

The graphical correlation between *x*_e_ and “Van’t Hoff model solubility (*x*^Van’t^)” of PPN is presented in Supplementary Materials Figure S1, which shows good graphical correlation. The root mean square deviations (*RMSDs*) for PPN in various “THP + water” combinations including pure water and pure THP were recorded as 0.31–1.11% with an average *RMSD* of 0.65%. In addition, the determination coefficients (*R*^2^) were obtained as 0.9935–0.9985. 

The results of the “Apelblat model” for PPN in different “THP + water” mixtures including pure water and pure THP are summarized in Table 4. 

**Figure 4:**
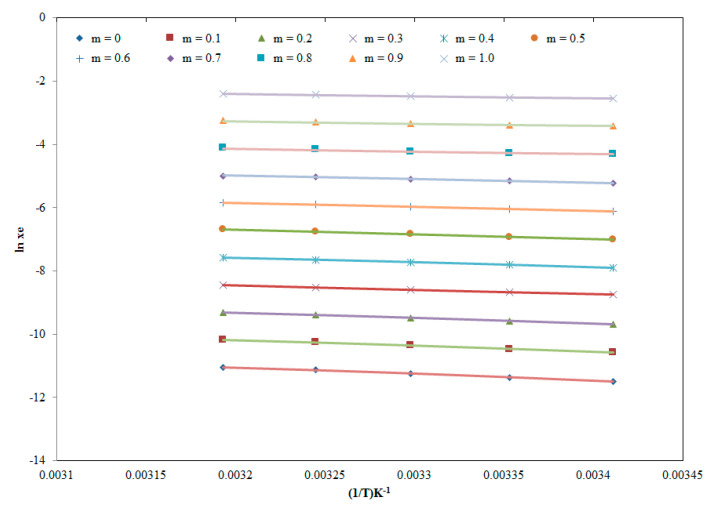
Correlation of experimental solubility values of PPN with “Apelblat model” in different “THP + water” mixtures at “*T* = 298.2–318.2 K”; Apelblat model solubility values of PPN are represented by solid lines, and experimental solubility values of PPN are represented by the symbols.

The graphical correlation between *x*_e_ and “Alelblat model solubility (*x*^Apl^)” values of PPN are presented in Figure 4, which expressed good graphical correlation. 

The *RMSD*s for PPN in various “THP + water” combinations including pure water and pure THP were estimated as 0.34–0.78% with an average *RMSD* of 0.54%. In addition, the *R*^2^ values were estimated as 0.9978–0.9999.

Results of the “Yalkowsky–Roseman model” for PPN in different “THP + water” combinations are listed in Table 5. The *RMSD* values for PPN in different “THP + water” combinations were computed as 0.46–2.81% with an average *RMSD* of 1.24%.

Results of the “Jouyban–Acree model” for PPN in “THP + water” mixtures are listed in Table 6. The average *RMSD* for PPN was estimated as 0.42%.

Results of the “Jouyban–Acree–Van’t Hoff model” for PPN in “THP + water” mixtures are tabulated in Table 6. The average *RMSD* for PPN was estimated as 0.54%.

As per the results recorded for solubility modeling, it was observed that all investigated models showed low *RMSD*s (average *RMSD* < 2.0%), which indicated good correlation of obtained solubility data of PPN with all investigated models. However, it should be noted that the error values of every model could not be compared with each other as each model was related with different parameters and model coefficients [49]. In general, the performance of all investigated models was good, but the “Jouyban–Acree model” could be considered as the most suitable model because it utilized the least number of model coefficients in addition to having a low *RMSD* value.

## 3. Experimental

### 3.1. Materials

PPN and THP were procured from “Beijing Mesochem Technology Co. Pvt. Ltd. (Beijing, China)” and “Gattefosse (Lyon, France)”, respectively. Water was collected from a Milli-Q water purification unit. The properties of materials are listed in Table 7.

### 3.2. PPN Solubility Measurement

A well-known saturation shake flask method was applied to measure the solubility of PPN in various “THP + water” combinations (*m* = 0.1–0.9) including pure water (*m* = 0.0) and pure THP (*m* = 1.0) [50]. This study was performed at “*T* = 298.2–318.2 K” and “*p* = 0.1 MPa”, and each study was repeated at least for three times. Excess PPN powder was taken into glass vials having 1.0 g of each “THP + water” mixtures including pure solvents. All the prepared samples were transferred to a “OLS 200 Grant Scientific Biological Shaker (Grant Scientific, Cambridge, UK)” after temperature and shaker speed settings. After equilibrium, the samples were removed from the shaker, centrifuged and diluted using methanol (mobile phase) and utilized for the determination of PPN content using the reported high-performance liquid chromatography method at 254 nm [38]. The amount of PPN in each sample was determined using a calibration curve of PPN. The *x*_e_ values of PPN were calculated using Equations (1) and (2) [26,27]:

(1) $$x_{e}=\frac{m_{1}/M_{1}}{m_{1}/M_{1}+m_{2}/M_{2}}$$

(2) $$x_{e}=\frac{m_{1}/M_{1}}{m_{1}/M_{1}+m_{2}/M_{2}+m_{3}/M_{3}}$$

Here, *m*_1_ = PPN mass; *m*_2_ = THP mass; *m*_3_ = water mass; *M*_1_ = PPN molar mass; *M*_2_ = THP molar mass and *M*_3_ = water molar mass. PPN solubility in pure water and pure THP was computed by applying Equation (1). PPN solubility in “THP + water” mixtures was calculated using Equation (2).

### 3.3. Computation of HSPs

If the solubility parameter of the solute is close to that of pure solvents or cosolvent mixtures, the solubility of solute will be higher in that particular pure solvent or cosolvent mixtures [51]. Therefore, HSPs for PPN, pure THP, pure water and various “THP + water” mixtures free of PPN were computed in this research. The *δ* value for PPN, pure THP and pure water was computed by applying Equation (3) [49,51,52] as follows:

(3) $$\delta^{2}=\delta_{d}^{2}+\delta_{p}^{2}+\delta_{h}^{2}$$

in which “*δ* = total HSP; *δ*_d_ = dispersion HSP; *δ*_p_ = polar HSP and *δ*_h_ = hydrogen-bonded HSP”. The HSPs for PPN, pure THP and pure water were estimated using “HSPiP software (version 4.1.07, Louisville, KY, USA)” [51]. The HSPs of various “THP + water” mixtures free of PPN (*δ*_mix_) were calculated using Equation (4) [26,53] as follows:

(4) $$\delta_{\mathrm{mix}}=\propto \delta_{1}+\left(1-\propto \right)\delta_{2}$$

Here, *α* = volume fraction of THP in “THP + water” mixtures; *δ*_1_ = HSP of pure THP and *δ*_2_ = HSP of pure water.

### 3.4. Mixing Thermodynamics Parameters of PPN Solution

Different mixing thermodynamic parameters of PPN solution were computed using the “Lewis–Randall rule”. For an ideal solution, different mixing thermodynamic parameters such as “mixing Gibbs energy (Δ_mix_*G*^id^), mixing entropy (Δ_mix_*S*^id^) and mixing enthalpy (Δ_mix_*H*^id^)” in different “THP + water” mixtures including pure water and pure THP can be calculated using Equations (5)–(7) [54,55] as follows:

(5) $$\Delta_{\mathrm{mix}}G^{\mathrm{id}}=RT\;\left(x_{1}\ln x_{1}+x_{2}\ln x_{2}+x_{3}\ln x_{3}\right)$$

(6) $$\Delta_{\mathrm{mix}}S^{\mathrm{id}}=-R\;\left(x_{1}\ln x_{1}+x_{2}\ln x_{2}+x_{3}\ln x_{3}\right)$$

(7) $$\Delta_{\mathrm{mix}}H^{\mathrm{id}}=0$$

Here, *R* = universal gas constant (*R* = 8.314 J/mol/K); *x*_1_ = PPN mole fraction; *x*_2_ = THP mole fraction and *x*_3_ = water mole fraction. 

For a non-ideal solution, various mixing thermodynamic parameters such as Δ_mix_*G*, Δ_mix_*H* and Δ_mix_*S* in different “THP + water” mixtures including pure water and pure THP can be calculated using Equations (8)–(10) [54,55,56] as follows:

(8) $$\Delta_{\mathrm{mix}}G=\Delta_{\mathrm{mix}}G^{\mathrm{id}}+G^{E}$$

(9) $$\Delta_{\mathrm{mix}}H=\Delta_{\mathrm{mix}}H^{\mathrm{id}}+H^{E}$$

(10) $$\Delta_{\mathrm{mix}}H=\Delta_{\mathrm{mix}}H^{\mathrm{id}}+H^{E}$$

Here, *G*^E^ = excess Gibbs energy and *H*^E^ = excess enthalpy. The *G*^E^ and *H*^E^ values were computed using the activity coefficient-based Wilson model by applying Equations (11) and (12) [56,57] as follows:

(11) $$G^{E}=RT\;\left(x_{1}\ln\gamma_{i}+x_{2}\ln\gamma_{i}+x_{3}\ln\gamma_{i}\right)$$

(12) $$H^{E}=-T^{2}\left[\partial \left(\frac{G^{E}/T}{\partial T}\right)\right]$$

The *γ*_i_ value for PPN in different THP + water combinations including pure water and pure THP was calculated by applying Equation (13) [58,59,60] as follows:

(13) $$\gamma_{i}=\frac{x^{\mathrm{idl}}}{x_{e}}$$

Here, *x*^idl^ = ideal solubility of PPN which was computed using Equation (14) [58] as follows:

(14) $$\ln\;x^{\mathrm{idl}}=\frac{-\Delta H_{\mathrm{fus}}\left(T_{\mathrm{fus}}-T\right)}{RT_{\mathrm{fus}}T}+\left(\frac{\Delta C_{p}}{R}\right)[\frac{T_{\mathrm{fus}}-T}{T}+\ln\left(\frac{T}{T_{\mathrm{fus}}}\right)]\;$$

Here, ∆*C*_p_ = difference between the molar heat capacity of solid state and liquid state; *T*_fus_ = fusion temperature of PPN and ∆*H*_fus_ = fusion enthalpy of PPN [59,61]. The values of *T*_fus_, ∆*H*_fus_ and ∆*C*_p_ for PPN were taken as 404.88 K, 32.69 kJ mol^−1^ and 80.74 J mol^−1^ K^−1^, respectively, from reference [38]. 

### 3.5. Solute–Solvent Molecular Interactions

The molecular interactions between PPN and various “THP + water” mixtures including pure water and pure THP can be explained using activity coefficients values. The *γ*_i_ values for PPN in different “THP + water” mixtures and pure solvents at “*T* = 298.2–318.2 K” were calculated by applying Equation (13) listed above. 

### 3.6. Thermodynamics-Based Computational Models

The solubility value obtained in the current research for PPN in various “THP + water” combinations including pure solvents was correlated using “Van’t Hoff, Apelblat, Yalkowsky–Roseman, Jouyban–Acree and Jouyban–Acree–Van’t Hoff” models [26,43,44,45,46,47,48]. The *x*^Van’t^ value of PPN in different “THP + water” mixtures including pure water and pure THP was calculated by applying Equation (15) [26] as follows:

(15) $$\ln\;x^{\mathrm{Va}n’t}=a+\frac{b}{T}$$

in which *a* and *b* = model coefficients of Equation (15), which were determined by the graphs constructed between ln **x*_e_* of PPN and 1/*T*. The correlation between *x*_e_ and *x*^Van’t^ values of PPN was carried out using *RMSD* and *R*^2^. The *RMSD*s of for PPN were calculated using its formula reported previously in the literature [27]. The *x*^Apl^ value of PPN in various “THP + water” combinations including pure water and pure THP was calculated using Equation (16) [43,44].

(16) $$\ln\;x^{\mathrm{Apl}}=A+\frac{B}{T}+C\ln\left(T\right)$$

Here, *A, B* and *C* = the model coefficients of Equation (16), which were obtained using “nonlinear multivariate regression analysis” of *x*_e_ values of PPN summarized in Table 1 [26]. The correlation between *x*_e_ and *x*^Apl^ values of PPN was again performed using *RMSD* and *R*^2^. The logarithmic solubility of “Yalkowsky–Roseman model (log *x*^Yal^)” for PPN in various “THP + water” mixtures was calculated by applying Equation (17) [45] as follows:

(17) $$\log x^{\mathrm{Yal}}=m_{1}logx_{1}+m_{2}logx_{2}$$

Here, *x*_1_ = mole fraction solubility of PPN in THP; *x*_2_ = mole fraction solubility of PPN in water; *m*_1_ = THP mass fraction and *m*_2_ = water mass fraction.

The “Jouyban–Acree model solubility (*x_m,T_*)” of PPN in different “THP + water” combinations was calculated by applying Equation (18) [62,63,64] as follows:

(18) $$\ln\;x_{m}{,}_{T}=m_{1}\ln x_{1}+m_{2}\;\ln\;x_{2}+[\frac{m_{1\;}m_{2}}{T}\overset{2}{\underset{i=0}{\sum }}\;J_{i}\left(m_{1}-m_{2}\right)]$$

Here, *J*_i_ = model coefficient of Equation (18) which was obtained using “no-intercept regression analysis” [65,66]. Based on the current data set, the trained version of Equation (18) can be expressed using Equation (19).

(19) $$\ln\;x_{m}{,}_{T}=m_{1}\ln x_{1}+m_{2\;}\ln\;x_{2}+\frac{-14.43m_{1}\;m_{2}}{T}$$

The correlation between *x*_e_ and *x_m,T_* of PPN was conducted using *RMSD*. The “Jouyban–Acree–Van’t Hoff model solubility of PPN (*x_m,T_*)” in different “THP + water” combinations was calculated by applying Equation (20) [26,66] as follows:

(20) $$\ln\;x_{m}{,}_{T}=m_{1}\left(A_{1}+\frac{B_{1}}{T}\right)+m_{2}\;\left(A_{2}+\frac{B_{2}}{T}\right)+\left[\frac{m_{1}m_{2}}{T}\;\overset{2}{\underset{i=0}{\sum }}J_{i}\left(m_{1}-m_{2}\right)\right]$$

Here, *A*_1_, *B*_1_, *A*_2_, *B*_2_ and *J_i_* = the model coefficient of Equation (20). Based on the current data set, the trained version of Equation (20) can be expressed using Equation (21).

(21) $$\ln\;x_{m}{,}_{T}=m_{1}\left(-0.21-\frac{-696.21}{T}\right)+m_{2}\;\left(-4.45-\frac{-2093.60}{T}\right)+\frac{-16.42m_{1}m_{2}}{T}$$

## 4. Conclusions

This study was aimed to determine the solubility of a bioactive compound PPN in various “THP + water” combinations including pure water and pure THP at “*T* = 298.2–318.2 K” and “*p* = 0.1 MPa”. The solubility of PPN was recorded as increasing with arise in both *m* value of THP and temperature in all “THP + water” mixtures including pure water and pure THP. Obtained solubility data of PPN was correlated well by “Apelblat, Van’t Hoff, Yalkowsky–Roseman, Jouyban–Acree and Jouyban–Acree–Van’t Hoff” models with an average *RMSD* of <2.0%. Overall, the “Jouyban–Acree model” was found as the most suitable for this modeling. The maximum solute–solvent interactions were observed in the PPN–THP combination in comparison to PPN–water. The results of mixing thermodynamics showed an endothermic dissolution of PPN at *m* = 0.6–1.0. In addition, the dissolution of PPN was found as entropy-driven at *m* = 0.8–1.0. 

## Figures and Tables

**Table 1:** Experimental solubilities (*x*_e_) of piperine (PPN) in mole fraction in different “Transcutol-HP (THP) + water” mixtures (*m*) at “*T* = 298.2–318.2 K” and “*p* = 0.1 MPa” ^a^.

*m*	*x*_e_				
	*T* = 298.2 K	*T* = 303.2 K	*T* = 308.2 K	*T* = 313.2 K	*T* = 318.2 K
0.0	1.03 × 10^−5^	1.17 × 10^−5^	1.31 × 10^−5^	1.47 × 10^−5^	1.59 × 10^−5^
0.1	2.57 × 10^−5^	2.85 × 10^−5^	3.19 × 10^−5^	3.55 × 10^−5^	3.80 × 10^−5^
0.2	6.20 × 10^−5^	6.88 × 10^−5^	7.61 × 10^−5^	8.40 × 10^−5^	9.01 × 10^−5^
0.3	1.59 × 10^−4^	1.71 × 10^−4^	1.86 × 10^−4^	1.99 × 10^−4^	2.15 × 10^−4^
0.4	3.71 × 10^−4^	4.07 × 10^−4^	4.42 × 10^−4^	4.79 × 10^−4^	5.09 × 10^−4^
0.5	9.06 × 10^−4^	9.80 × 10^−4^	1.08 × 10^−3^	1.16 × 10^−3^	1.25 × 10^−3^
0.6	2.23 × 10^−3^	2.39 × 10^−3^	2.56 × 10^−3^	2.74 × 10^−3^	2.88 × 10^−3^
0.7	5.40 × 10^−3^	5.74 × 10^−3^	6.10 × 10^−3^	6.51 × 10^−3^	6.80 × 10^−3^
0.8	1.35 × 10^−2^	1.40 × 10^−2^	1.47 × 10^−2^	1.55 × 10^−2^	1.63 × 10^−2^
0.9	3.26 × 10^−2^	3.37 × 10^−2^	3.53 × 10^−2^	3.70 × 10^−2^	3.87 × 10^−2^
1.0	7.88 × 10^−2^	8.12 × 10^−2^	8.44 × 10^−2^	8.79 × 10^−2^	9.10 × 10^−2^
*x* ^idl^	5.13 × 10^−2^	6.02 × 10^−2^	7.06 × 10^−2^	8.26 × 10^−2^	9.63 × 10^−2^

^a^ The relative uncertainties *u*_r_ are *u*_r_(*T*) = 0.010, *u*_r_(*m*) = 0.001%, *u*(*p*) = 0.003 and *u*_r_(*x*_e_) = 0.11.

**Table 2:** Activity coefficients (*γ_i_*) of PPN in different “THP + water” mixtures (*m*) at “*T* = 298.2–318.2 K”.

*m*	*γ*_i_				
	*T* = 298.2 K	*T* = 303.2 K	*T* = 308.2 K	*T* = 313.2 K	*T* = 318.2 K
0.0	4980.00	5150.00	5380.00	5620.00	6050.00
0.1	1995.20	2108.92	2215.74	2339.59	2533.27
0.2	827.00	875.00	927.00	984.00	1070.00
0.3	322.00	353.00	380.00	416.00	448.00
0.4	138.00	148.00	160.00	173.00	189.00
0.5	56.60	61.40	65.50	71.40	77.30
0.6	23.00	25.20	27.60	30.20	33.40
0.7	5.40	5.74	6.10	6.51	6.80
0.8	3.81	4.31	4.82	5.33	5.92
0.9	1.57	1.79	2.00	2.23	2.49
1.0	0.65	0.74	0.83	0.94	1.06

**Table 3:** Results of “Van’t Hoff model” for PPN in “THP + water” combinations (*m*) ^b^.

*m*	*a*	*b*	*R* ^2^	*RMSD* (%)	Overall *RMSD* (%)
0.0	−4.45	−2093.60	0.9960	1.11	
0.1	−4.20	−1897.30	0.9963	0.91	
0.2	−3.65	−1799.00	0.9973	0.70	
0.3	−3.98	−1421.50	0.9982	0.33	
0.4	−2.83	−1509.30	0.9968	0.62	
0.5	−1.90	−1520.50	0.9981	0.77	0.65
0.6	−1.95	−1238.70	0.9985	0.42	
0.7	−1.49	−1112.00	0.9973	0.42	
0.8	−1.24	−916.75	0.9935	0.56	
0.9	−0.64	−829.34	0.9932	1.01	
1.0	−0.21	−696.21	0.9960	0.31	

^b^ The average relative uncertainties are *u*(*a*) = 0.30 and *u*(*b*) = 0.07.

**Table 4:** Results of “Apelblat model” for PPN in “THP + water” combinations (*m*) ^c^.

*m*	*A*	*B*	*C*	*R* ^2^	*RMSD* (%)	Overall *RMSD* (%)
0.0	331.19	−17,505.00	−49.84	0.9995	0.78	
0.1	224.66	−12,407.50	−33.98	0.9982	0.73	
0.2	217.93	−11,974.50	−32.90	0.9993	0.58	
0.3	−105.09	3214.87	15.01	0.9988	0.57	
0.4	228.14	−12,114.70	−34.29	0.9999	0.60	
0.5	45.42	−3697.43	−7.02	0.9981	0.45	0.54
0.6	87.58	−5351.77	−13.29	0.9991	0.34	
0.7	84.34	−5054.78	−12.74	0.9981	0.44	
0.8	−157.86	6268.79	23.26	0.9978	0.61	
0.9	−157.97	6388.73	23.36	0.9985	0.45	
1.0	−84.70	3179.61	12.54	0.9982	0.47	

^c^ The average relative uncertainties are *u*(*A*) = 0.92, *u*(*B*) = 1.54 and *u*(*C*) = 0.90.

**Table 5:** Results of “Yalkowsky–Roseman model” for PPN in different “THP + water” mixtures (*m*) at “*T* = 298.2–318.2 K”.

*m*	Log *x*^Yal^					*RMSD* (%)	Overall *RMSD* (%)
	*T* = 298.2 K	*T* = 303.2 K	*T* = 308.2 K	*T* = 313.2 K	*T* = 318.2 K		
0.1	−4.59	−4.54	−4.50	−4.45	−4.42	1.21	
0.2	−4.21	−4.16	−4.12	−4.07	−4.04	0.46	
0.3	−3.82	−3.77	−3.74	−3.69	−3.67	2.81	
0.4	−3.43	−3.39	−3.35	−3.32	−2.29	0.91	
0.5	−3.04	−3.01	−2.97	−2.94	−2.91	2.27	1.24
0.6	−2.65	−2.62	−2.59	−2.56	−2.54	1.11	
0.7	−2.26	−2.24	−2.21	−2.18	−2.16	0.38	
0.8	−1.88	−1.85	−1.83	−1.81	−1.79	1.31	
0.9	−1.49	−1.47	−1.45	−1.43	−1.41	0.78	

**Table 6:** Results of “Jouyban–Acree” and “Jouyban–Acree–Van’t Hoff” models for PPN in “THP + water” combinations.

System	Jouyban–Acree	Jouyban–Acree–Van’t Hoff
		*A*_1_–0.21
PEG-400 + water	*J*_i_–14.43	*B*_1_–696.21
		*A*_2_–4.45
		*B*_2_–2093.60
*RMSD* (%)	0.42	*J*_i_–16.42


		0.54

**Table 7:** List of materials used.

Material	Molecular Formula	Molar Mass (g mol^−1^)	CAS Registry No.	Purification Method	Mass Fraction Purity	Analysis Method	Analysis Method	Source
PPN	C_17_H_19_NO_3_	285.34	94-62-2	None	>0.99	HPLC	HPLC	Sigma Aldrich
THP	C_6_H_14_O_3_	134.17	111-90-0	None	>0.99	GC	GC	Gattefosse
Water	H_2_O	18.07	7732-18-5	None	-	-	-	Milli-Q

Purity and method of analysis were provided by supplier of each material.

## Supplementary Materials

The following are available online, Figure S1: Correlation of experimental solubility values of PPN with “Van’t Hoff model” in different “THP + water” mixtures at “*T* = 298.2–318.2 K”; Van’t Hoff model solubility values of PPN are represented by solid lines, and experimental solubility values of PPN are represented by the symbols, Table S1: Hansen solubility parameters (*δ*_mix_/MPa^1/2^) for various THP + water mixtures free of PPN at “*T* = 298.2 K”, Table S2: The values of mixing enthalpy (Δ_mix_*H*/J mol^−1^), mixing entropy (Δ_mix_*S*/J mol^−1^ K^−1^), mixing Gibbs energy (Δ_mix_*G*/J mol^−1^) and activity coefficient (*γ*_i_) for PPN dissolution in different “THP + water” mixtures.
