# Supplementary material for: Solubility Data of the Bioactive Compound Piperine in (Transcutol + Water) Mixtures: Computational Modeling, Hansen Solubility Parameters and Mixing Thermodynamic Parameters
Source: Molecules. 2020 Jun 13;25(12):2743. doi: 10.3390/molecules25122743 (PMC7355804; doi:10.3390/molecules25122743)
Supplement: Supplementary file 1 [file molecules-25-02743-s001.pdf]

Solubility Data of the Bioactive Compound Piperine in (Transcutol + Water) Mixtures: Computational Modeling, Hansen Solubility Parameters and Mixing Thermodynamic Parameters

Faiyaz Shakeel, Nazrul Haq and Sultan Alshehri*

Department of Pharmaceutics, College of Pharmacy, King Saud University, P.O. Box 2457, Riyadh 11451, Saudi Arabia

***Corresponding Author:**

Dr. Sultan Alshehri,

Department of Pharmaceutics, College of Pharmacy,

King Saud University, Riyadh, Saudi Arabia

Email: aslshehri@ksu.edu.sa

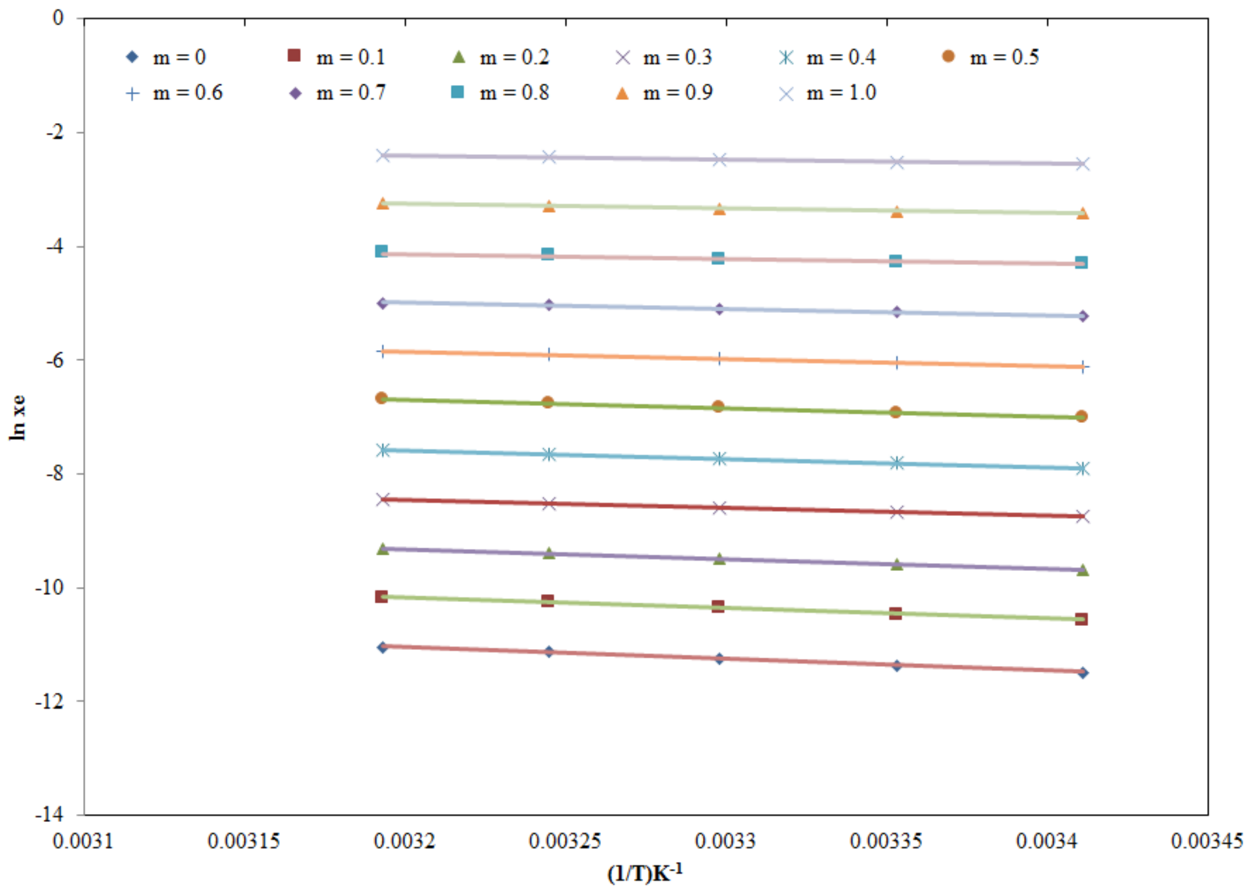

Figure S1. Correlation of experimental solubility values of PPN with Van't Hoff model in different THP + water mixtures at T = 298.2 K to 318.2 K; Van't Hoff model solubility values of PPN are represented by solid lines, and experimental solubility values of PPN are represented by the symbols.

Table S1. Hansen solubility parameters (δ_{mix}/MPa$^{1/2}$) for various THP + water mixtures free of PPN at T = 298.2 K

m	δ_{mix}/MPa$^{1/2}$
0.1	45.16
0.2	42.52
0.3	39.88
0.4	37.24
0.5	34.60
0.6	31.96
0.7	29.32
0.8	26.68
0.9	24.04

Table S2. The values of mixing enthalpy ($\Delta_{mix}H$/J mol^{-1}), mixing entropy ($\Delta_{mix}S$/J mol^{-1} K^{-1}), mixing Gibbs energy ($\Delta_{mix}G$/J mol^{-1}) and activity coefficient (γ_i) for PPN dissolution in different THP + water mixtures[a].

T/K	$\Delta_{mix}H$/J mol^{-1}	$\Delta_{mix}G$/J mol^{-1}	$\Delta_{mix}S$/J mol^{-1} K^{-1}	γ_i
m = 0.0				
298.2	–1170	774	–6.52	4980
303.2	–1200	791	–6.55	5150
308.2	–1220.	811	–6.60	5380
313.2	–1250	830	–6.63	5620
318.2	–1280	854	–6.70	6050
m = 0.1				
298.2	–955	571	–5.12	1995
303.2	–978	587	–5.16	2108
308.2	–1000	603	–5.20	2215
313.2	–1020	620	–5.25	2329
318.2	–1050	641	–5.32	2533
m = 0.2				
298.2	–764	397	–3.89	827
303.2	–784	411	–3.94	875
308.2	–803	424	–3.98	927
313.2	–824	438	–4.02	984
318.2	–847	455	–4.09	1070
m = 0.3				
298.2	–589	242	–2.78	322
303.2	–608	255	–2.84	353
308.2	–626	267	–2.89	380
313.2	–646	281	–2.96	416
318.2	–665	294	–3.01	448
m = 0.4				
298.2	–443	119	–1.88	138
303.2	–457	128	–1.93	148
308.2	–472	137	–1.97	160
313.2	–487	146	–2.02	173
318.2	–503	157	–2.07	189
m = 0.5				
298.2	–316	16	–1.11	56
303.2	–328	23	–1.15	61
308.2	–338	28	–1.19	65
313.2	–351	36	–1.23	71
318.2	–363	43	–1.27	77
m = 0.6				
298.2	–208	–63	–0.48	23
303.2	–217	–58	–0.52	25
308.2	–227	–52	–0.56	27
313.2	–237	–47	–0.60	30
318.2	–248	–41	–0.65	33
m = 0.7				
298.2	–123	–117	–0.02	9
303.2	–131	–114	–0.05	10
308.2	–138	–110	–0.09	11
313.2	–146	–107	–0.12	12
318.2	–155	–102	–0.16	14

m = 0.8				
298.2	–57	–147	0.30	3
303.2	–63	–144	0.26	4
308.2	–70	–142	0.23	5
313.2	–75	–140	0.20	5
318.2	–81	–137	0.17	6
m = 0.9				
298.2	–14	–149	0.45	1.57
303.2	–18	–147	0.42	1.79
308.2	–22	–146	0.40	2.00
313.2	–26	–145	0.37	2.23
318.2	–30	–144	0.35	2.49
m = 1.0				
298.2	8	–111	0.40	0.65
303.2	6	–110	0.38	0.74
308.2	4	–110	0.36	0.83
313.2	1	–110	0.35	0.94
318.2	1	–110	0.34	1.06

[a]The average relative uncertainties are $u(\Delta_{mix}H)$ = 0.90, $u(\Delta_{mix}G)$ = 2.04 and $u(\Delta_{mix}S)$ = 1.19.
